# Antifungal mechanism of *Bacillus amyloliquefaciens* strain GKT04 against *Fusarium* wilt revealed using genomic and transcriptomic analyses

**DOI:** 10.1002/mbo3.1192

**Published:** 2021-05-24

**Authors:** Dandan Tian, Xiupeng Song, Chaosheng Li, Wei Zhou, Liuyan Qin, Liping Wei, Wei Di, Sumei Huang, Baoshen Li, Quyan Huang, Shengfeng Long, Zhangfei He, Shaolong Wei

**Affiliations:** ^1^ Biotechnology Research Institute Guangxi Academy of Agricultural Sciences Nanning China; ^2^ Sugarcane Research Institute Guangxi Academy of Agricultural Sciences Nanning China

**Keywords:** Antifungal, *Bacillus amyloliquefaciens*, *Fusarium* Wilt, transcriptome, whole‐genome sequencing

## Abstract

The application of endophytic bacteria, particularly members of the genus *Bacillus*, offers a promising strategy for the biocontrol of plant fungal diseases, owing to their sustainability and ecological safety. Although multiple secondary metabolites that demonstrate antifungal capacity have been identified in diverse endophytic bacteria, the regulatory mechanisms of their biosynthesis remain largely unknown. To elucidate this, we sequenced the entire genome of *Bacillus amyloliquefaciens* GKT04, a strain isolated from banana root, which showed high inhibitory activity against *Fusarium oxysporum* f. sp. cubense race 4 (FOC4). The GKT04 genome consists of a circular chromosome and a circular plasmid, which harbors 4,087 protein‐coding genes and 113 RNA genes. Eight gene clusters that could potentially encode antifungal components were identified. We further applied RNA‐Seq analysis to survey genome‐wide changes in the gene expression of strain GKT04 during its inhibition of FOC4. In total, 575 upregulated and 242 downregulated genes enriched in several amino acid and carbohydrate metabolism pathways were identified. Specifically, gene clusters associated with difficidin, bacillibactin, and bacilysin were significantly upregulated, and their gene regulatory networks were constructed. Our work thereby provides insights into the genomic features and gene expression patterns of this *B*. *amyloliquefaciens* strain, which presents an excellent potential for the biocontrol of *Fusarium* wilt.

## Graphical abstract

The genome of *Bacillus amyloliquefaciens* GKT04, a strain that showed high inhibitory activity against *Fusarium* wilt, harbors 4,087 protein‐coding genes and 113 RNA genes, including eight gene clusters that potentially encode antifungal components. Among those 575 upregulated and 242 downregulated genes were identified from transcriptome during the inhibition of *Fusarium oxysporum* f. sp. cubense race 4 of GKT04. The gene clusters associated with difficidin, bacillibactin, and bacilysin were significantly upregulated, and the corresponding gene regulatory networks were constructed.

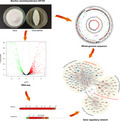

## 1 INTRODUCTION

Banana is a popular fruit crop that is widely cultivated in tropical and subtropical regions. *Fusarium* wilt of banana (*Musa* spp.), caused by *Fusarium oxysporum* f. sp. cubense race 4 (FOC4), is one of the most serious fungal diseases that limit worldwide sustainable banana production (Ploetz, 2006). FOC4 causes vascular necrosis in infected plants and thus causes a destructive reduction in banana crop output. Management of this disease is largely restricted to the exclusion of pathogens from non‐infested areas and the use of disease‐resistant varieties in areas where FOCs have been established. The perennial yield of this crop and the multi‐cycle nature of this disease limit the development of other management strategies. Although biological, chemical, and cultural measures effective against annual or short‐lived hosts of these diseases are usually ineffective against *Fusarium* wilt of banana, biocontrol has received considerable attention owing to its efficacy and environmental friendliness (Ploetz, 2015). According to Fravel et al. (Fravel et al., 2003), the difficulty in controlling *Fusarium* wilt has stimulated research on its biological control, rather than environmental protection concerns.

Endophytic bacteria, which colonize internal plant tissues without causing apparent harm to the host, are widespread (Senthilkumar et al., 2011). These bacteria are less susceptible to the influence of the external environment and can also inhibit the growth and invasion of pathogenic bacteria and prevent various plant diseases via multiple mechanisms, including the production of a variety of antibiotics. Thus, they are regarded as resources for the biocontrol of crops (Xia et al., 2015).

Studies have shown that *Fusarium* wilt is inhibited by a range of biocontrol endophytic bacteria, including *Pseudomonas aeruginosa* (Anjaiah et al., 2003), *Serratia marcescens* (Tan et al., 2015), and *Bacillus* sp. (Nam et al., 2009). *Bacillus* spp. can produce various antifungal components. A bio‐organic fertilizer enriched with *B*. *amyloliquefaciens* strain NJN‐6 suppressed *Fusarium* wilt in banana plants (Yuan et al., 2012). Wang et al. (Wang et al., 2016) discovered that *B*. *amyloliquefaciens* strain W19 suppressed *Fusarium* wilt in bananas under greenhouse and field conditions. These findings point to the enormous potential of *Bacillus* spp. in the management of *Fusarium* wilt.

Multiple secondary metabolites with antifungal capacity have been discovered in recent decades. Among them, antifungal compounds synthesized by non‐ribosomal polypeptide synthetase (NRPS) and polyketide synthetase (PKS) have frequently been identified in *Bacillus* spp. (Fira et al., 2018). The NRPS includes a versatile family of secondary metabolites such as siderophores, surfactants, pigments, and lipopeptides (Wang et al., 2014). Iturins and fengycins are two common lipopeptides from *Bacillus* spp. that are widely known for their strong antifungal activity against several plant fungi (Ongena & Jacques, 2008; Raaijmakers et al., 2010). They inhibit fungal growth by targeting cell walls and membranes. Siderophore is another thiotemplate NRPS whose mode of action differs from that of lipopeptides. Siderophores in *Bacillus* spp., including itoic acid and bacillibactin, chelate iron and reduce its bioavailability, thus antagonizing the growth of other surrounding microbes, such as *Fusarium*, by limiting their access to iron (Yu et al., 2011). The PKSs also comprise several compounds, such as bacillaene and macrolactins, whose antimicrobial activities against *Fusarium* spp. have been validated (Um et al., 2013; Yuan et al., 2012). However, the regulatory mechanisms underlying antifungal compound synthesis remain largely unknown.

The endophytic bacterium *B*. *amyloliquefaciens* strain GKT04 inhibits the growth and reproduction of FOC4 (Tian et al., 2018). Although the two lipopeptides, fengycins and bacillomycin, isolated from GKT04 have been shown to inhibit FOC4, the existence of other potential antifungal components in GKT04 remains unclear (Tian et al., 2020). Here, we combined the analyses of genome and transcriptome sequences to obtain additional information on the antifungal mechanism of *B*. *amyloliquefaciens* strain GKT04 at the omics level. Multiple genes involved in the synthesis of antibiotic metabolites, including polyketides, siderophores, and lipopeptides, were identified in the genome of this strain. Based on transcriptome sequence analysis, a series of genes encoding polyketide difficidin, siderophore bacillibactin, and the lipopeptide bacilysin were found to be upregulated in response to FOC4. Furthermore, a gene regulatory network for the biosynthesis of the antifungal metabolites difficidin, bacillibactin, and bacilysin was also present in this strain. These results allow us to better understand the antifungal metabolites and regulatory mechanisms involved in the synthesis of antifungal compounds in *B*. *amyloliquefaciens*.

## 2 MATERIALS AND METHODS

### 2.1 Bacterial strain culture and assessment of antifungal effect against *Fusarium* wilt

*B*. *amyloliquefaciens* strain GKT04 was previously isolated at the Guangxi Academy of Agricultural Sciences, Nanning, China, from the roots of the banana variety Guijiao 9, which is tolerant to *Fusarium* wilt. This bacterial strain was cultivated in LA medium at 30°C. To determine its antifungal activity against *Fusarium* wilt, the strain GKT04 was combined with FOC4 in potato dextrose agar (PDA) medium as previously described (Sun et al., 2010). In brief, FOC4 was placed at the center of a PDA plate, and the GKT04 colony was placed approximately 3 cm from FOC4. Plates were incubated for 5 days at 28°C, and the inhibition of fungal growth was monitored by recording the diameter of the inhibition zone (in millimeters). A PDA plate culture containing only FOC4 was used as a control. The percentage of growth inhibition by GKT04 was recorded on days 3 and 6 after incubation.

### 2.2 Genomic DNA extraction and whole‐genome sequencing

Genomic DNA was extracted from GKT04 by using the cetyltrimethylammonium bromide method (Wu et al., 1999). The quality and integrity of the genomic DNA were assessed using 0.8% agarose gel electrophoresis, with a single band of approximately 50 kb representing high‐quality DNA. DNA concentration and purity were measured using a NanoDrop 2000 spectrophotometer (Thermo Fisher Scientific, USA) and a Qubit 3.0 fluorometer (Thermo Fisher Scientific). For PacBio sequencing, genomic DNA was sheared into 20 kb fragments by using a Covaris g‐TUBE shearing device and then purified using AMPure XP beads (Beckman Coulter, USA). Fragmented genomic DNA was used for library construction by using the PacBio SMRTbell library preparation kit, according to the manufacturer's instructions. The SMRTbell libraries were then selected using BluePippin (Sage Science, Beverly, MA, USA) and sequenced on the PacBio RS II platform by BIOMARKER Ltd. (Beijing, China).

### 2.3 Genomic sequence analysis

The PacBio long reads were assembled using the Canu software (Koren et al., 2017). The completeness of the genomic assembly was assessed using BUSCO (Simão et al., 2015). Pairwise average nucleotide identity (ANI) between the GKT04 genome and other *B*. *amyloliquefaciens* genomes available from the NCBI database was analyzed using Pyani software (https://github.com/widdowquinn/pyani) with default parameters. The GKT04 genome was annotated using the Prodigal software (Hyatt et al., 2010). Protein‐coding genes were further annotated in other public databases, including NR (non‐redundant), Swiss‐Prot, Kyoto Encyclopedia of Genes and Genomes (KEGG), TrEMBL, Clusters of Orthologous Groups (COG), Gene Ontology (GO), and Pfam. Furthermore, the web‐service antiSMASH was used to identify potential antifungal proteins in the genome of GKT04 (Blin et al., 2019).

### 2.4 Total RNA extraction and RNA‐Seq

To obtain samples for RNA‐Seq, the strain GKT04 was cultured in PDA medium alone (CK) or in PDA medium combined with FOC4 as described above for 3 days (treatment). Total RNA was extracted from bacterial plaques from both cultures by using a Bacterial RNA Kit (Omega Bio‐Tek, Salt Lake City, UT, USA), according to the manufacturer's instructions. Sequencing libraries were constructed from three replicates of both samples and sequenced by GENE DENOVO, Ltd. (Guangzhou, China) on the Illumina HiSeq platform, producing 150 base paired‐end reads.

### 2.5 Differential gene expression analysis

All clean RNA‐Seq reads were aligned to the GKT04 genome sequence by using Bowtie2 (Langmead & Salzberg, 2012), and the fragments per kilobase per million (FPKM) method was used to describe gene expression (Mortazavi et al., 2008). Differentially expressed genes (DEGs) in treated samples versus CK were determined using the RSEM software (Li & Dewey, 2011), based on log_2_ fold change ≥1 and false discovery rate (FDR) ≤0.05 thresholds (Benjamini and Hochberg adjustment). The GO category and KEGG pathway enrichment of DEGs were determined by comparison with the annotation of total genes (adjusted *P*‐value ≤0.05, Bonferroni test). The protein–protein interaction (PPI) network of DEGs was analyzed using STRING (Szklarczyk et al., 2017) and visualized using Cytoscape software (Shannon et al., 2003).

## 3 RESULTS

### 3.1 In vitro antifungal activity of *B. amyloliquefaciens* GKT04

**FIGURE 1:**
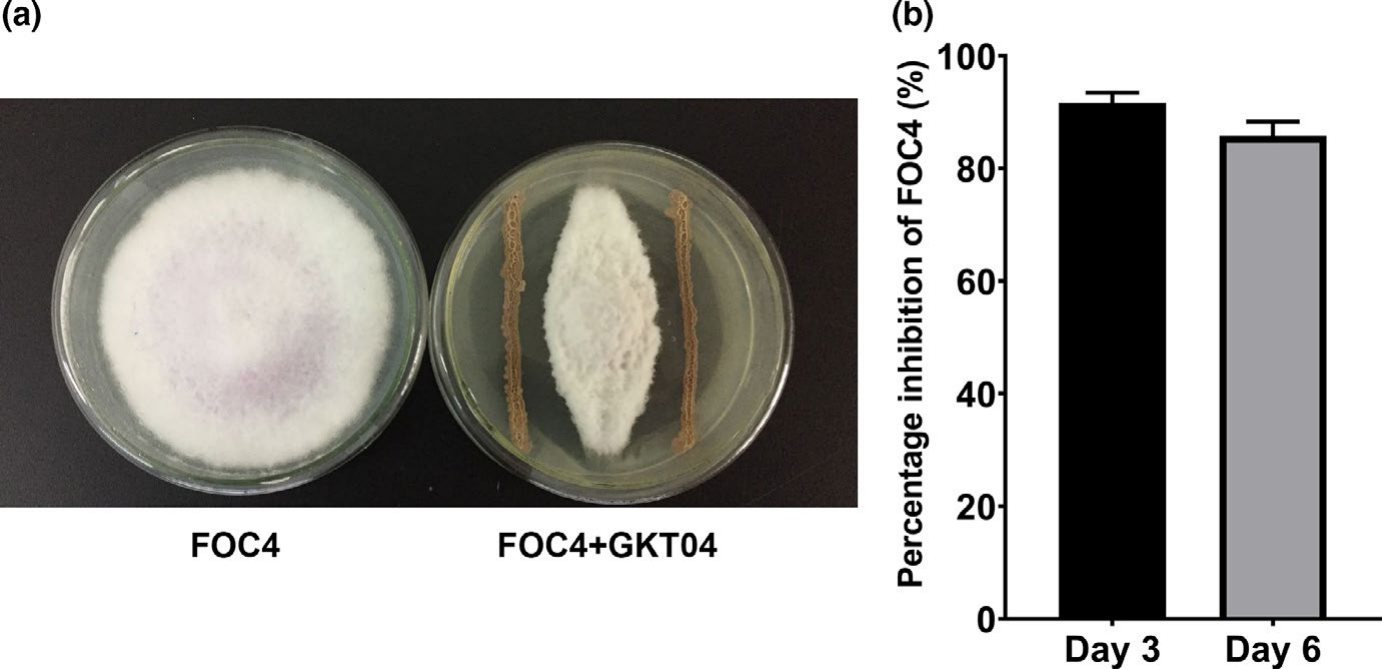
Inhibition of FOC4 growth by *B*. *amyloliquefaciens* strain GKT04. (a) Inhibition effect of strain GKT04 against FOC4 in in vitro assays. (b) Percentage of inhibition of FOC4 growth by strain GKT04 after 3 and 6 days of incubation. Data are shown as mean ± *SEM* (*n* = 3)

*Bacillus amyloliquefaciens* GKT04 colony was transferred to a PDA plate for evaluation of its in vitro antifungal activity. GKT04 showed a significant ability to suppress the growth of FOC4 when the two were co‐cultivated in vitro (Figure 1A). On Day 3 of the experiment, GKT04 showed strong inhibitory activity (91.67%, Figure 1B). The percentage of growth inhibition then decreased, but only slightly, to 85.72% by Day 6. These data illustrate that this bacterial strain has high antifungal activity.

### 3.2 Genomic features of *B. amyloliquefaciens* GKT04

**FIGURE 2:**
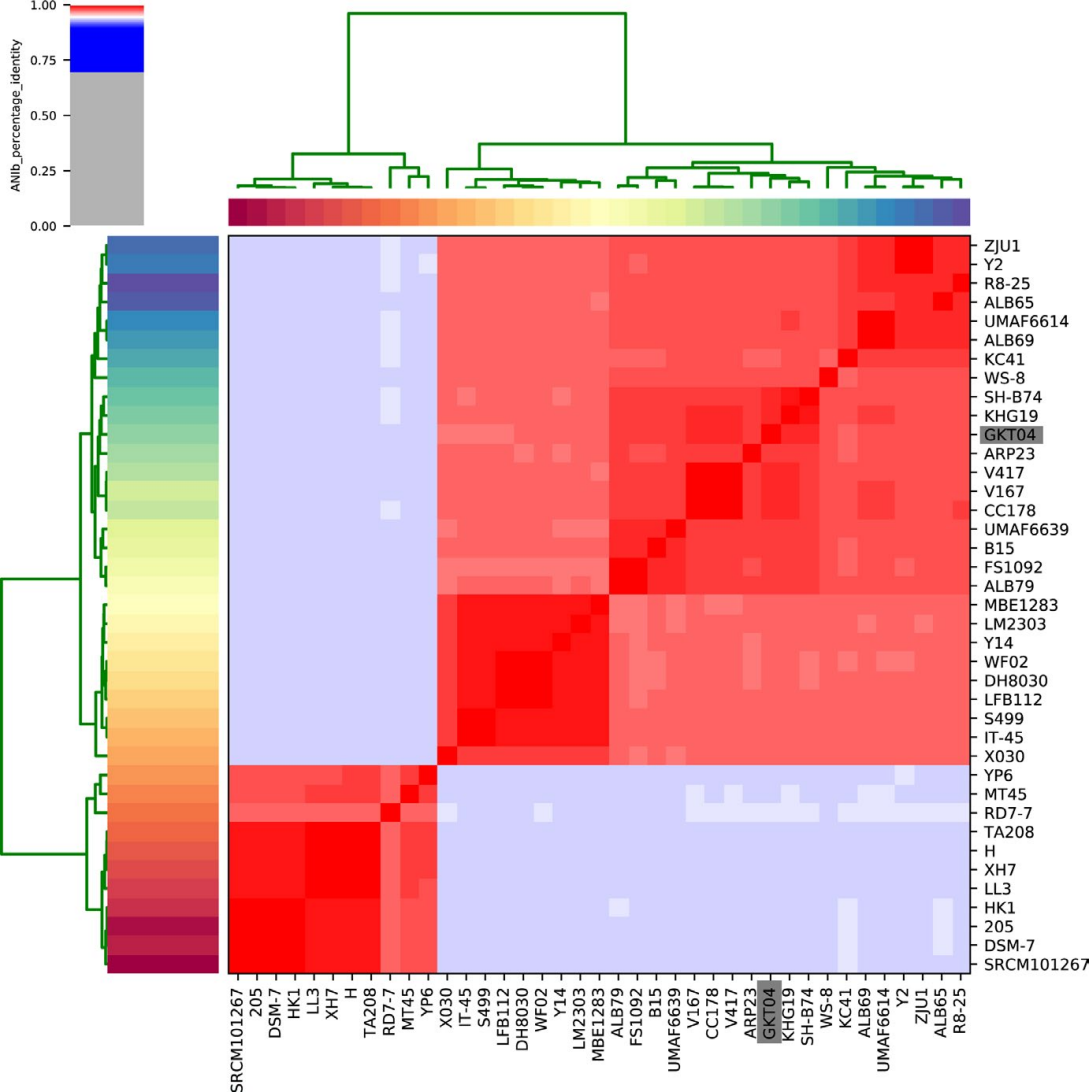
Pairwise average nucleotide identity (ANI) comparison of strain GKT04 and other *B*. *amyloliquefaciens* strains. Strain GKT04 is marked with a gray box

The complete genome sequence of *B*. *amyloliquefaciens* GKT04 was found to contain one chromosome that is 4,056,188 bp long and one plasmid that is 93,502 bp long. The average GC content was 46.39% in the chromosome and 41.48% in the plasmid. ANI analysis revealed that GKT04 shared an average ANI value of 97% with other *B*. *amyloliquefaciens* strains and was highly homologous to strains KHG19 and SH‐B74 (ANI value >98%) (Figure 2). A total of 3,936 protein‐coding genes, 27 rRNAs, and 86 tRNAs were detected in the chromosome, and 151 protein‐coding genes were detected in the plasmid.

### 3.3 Functional characterization of strain GKT04

**FIGURE 3:**
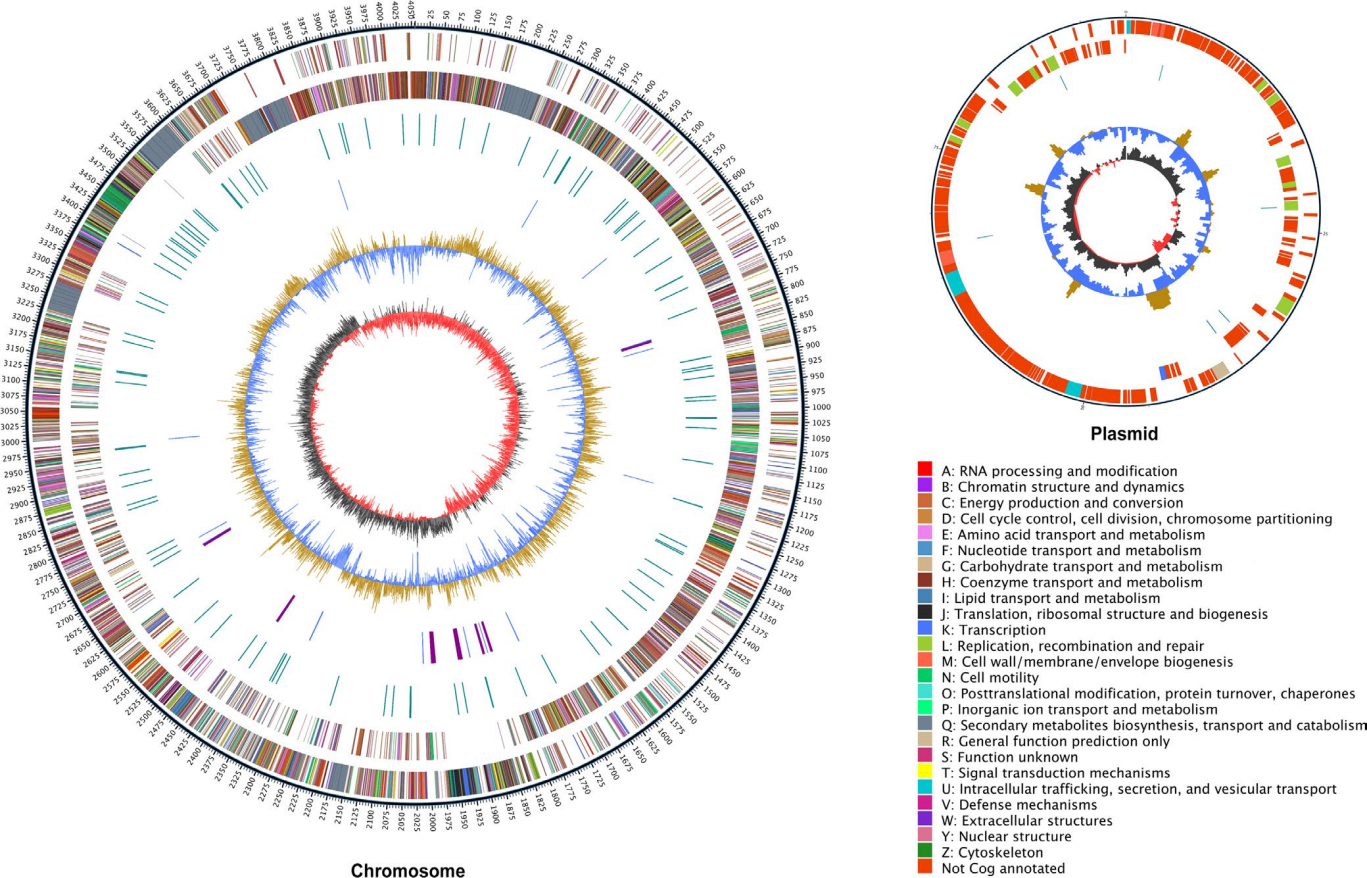
Circular representation of *B*. *amyloliquefaciens* GKT04 genome. The outer ring represents the genome scale. Ring 2 and ring 3 represent the protein‐coding genes at plus and minus strands, respectively, and different colors represent different COG categories of the corresponding genes. Ring 4 represents repeated sequences, and ring 5 represents structural RNAs. Ring 6 (black circle) represents the GC content (%), and the inner ring depicts the GC skew

**FIGURE A1:**
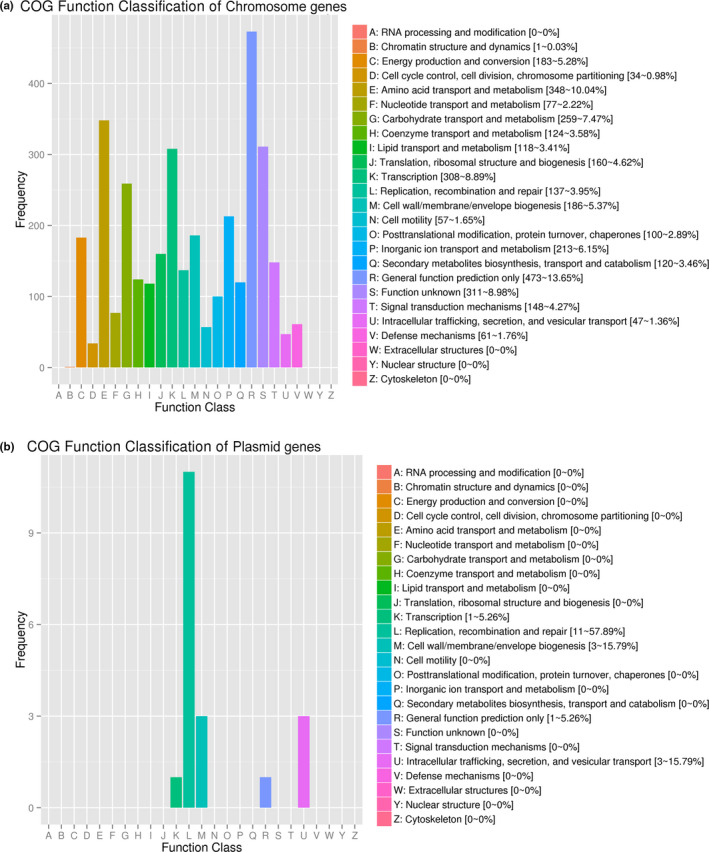
COG functional classification of protein‐encoding genes in the chromosome (a) and plasmid (b) of *B*. *amyloliquefaciens* GKT04.

**FIGURE A2:**
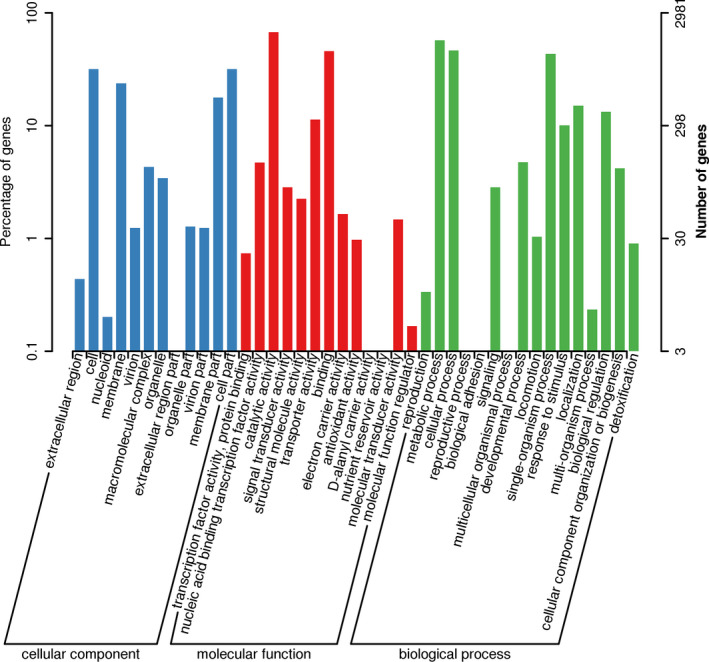
Gene Ontology (GO) functional categories of protein‐encoding genes in *B*. *amyloliquefaciens* GKT04

Protein annotation statistics are presented in Table 1. In total, 2,964 proteins were annotated to at least one COG functional category (Figure 3). Most proteins with known functions were associated with amino acid transport and metabolism, transcription, carbohydrate transport and metabolism, inorganic ion transport and metabolism, and cell wall/membrane/envelope biogenesis (Figure A1). GO annotation revealed that most of these genes were assigned to the functional categories of metabolic processes, cellular processes, catalytic activity, and binding (Figure A2). These functional categories indicate that this bacterial strain has a strong metabolic capacity. Further annotation in antiSMASH revealed that strain GKT04 might harbor multiple gene clusters that encode polyketides, siderophores, and lipopeptides such as difficidin, bacillibactin, bacilysin, surfactin, plantazolicin, macrolactin H, bacillaene, bacillomycin D, and fengycin (Table 2). Most functional genes and all gene clusters were located on the chromosome, and the plasmid comprised largely of genes with unknown functions.

**TABLE 1:** Functional annotation of genes in *B*. *amyloliquefaciens* GKT04.

Database	Annotated number in chromosome	Annotated number in plasmid	Total annotated number
COG	2,945	19	2,964
GO	2,981	18	2,999
KEGG	2,078	9	2,087
Pfam	3,425	28	3,453
Swiss‐Prot	3,595	37	3,632
TrEMBL	3,926	118	4,044
NR	3,927	121	4,048
All_Annotated	3,927	121	4,048

**TABLE 2:** Gene clusters involved in the biosynthesis of secondary metabolites in *B*. *amyloliquefaciens* GKT04 as predicted by antiSMASH.

Region	Type	From	To	Potential production
Region 1	transAT‐PKS‐like, transAT‐PKS	157,071	262,821	difficidin
Region 2	NRPS, bacteriocin	897,994	948,500	bacillibactin
Region 3	other	1,474,444	1,515,862	bacilysin
Region 4	NRPS	2,130,203	2,195,146	surfactin
Region 5	LAP	2,518,757	2,540,939	plantazolicin
Region 6	transAT‐PKS	3,202,936	3,290,769	macrolactin H
Region 7	transAT‐PKS, T3PKS, NRPS	3,511,459	3,621,086	bacillaene
Region 8	NRPS, transAT‐PKS, betalactone	3,688,879	3,826,702	fengycin/bacillomycin D

### 3.4 Transcriptomic profiles of strain GKT04 during FOC4 inhibition

**FIGURE 4:**
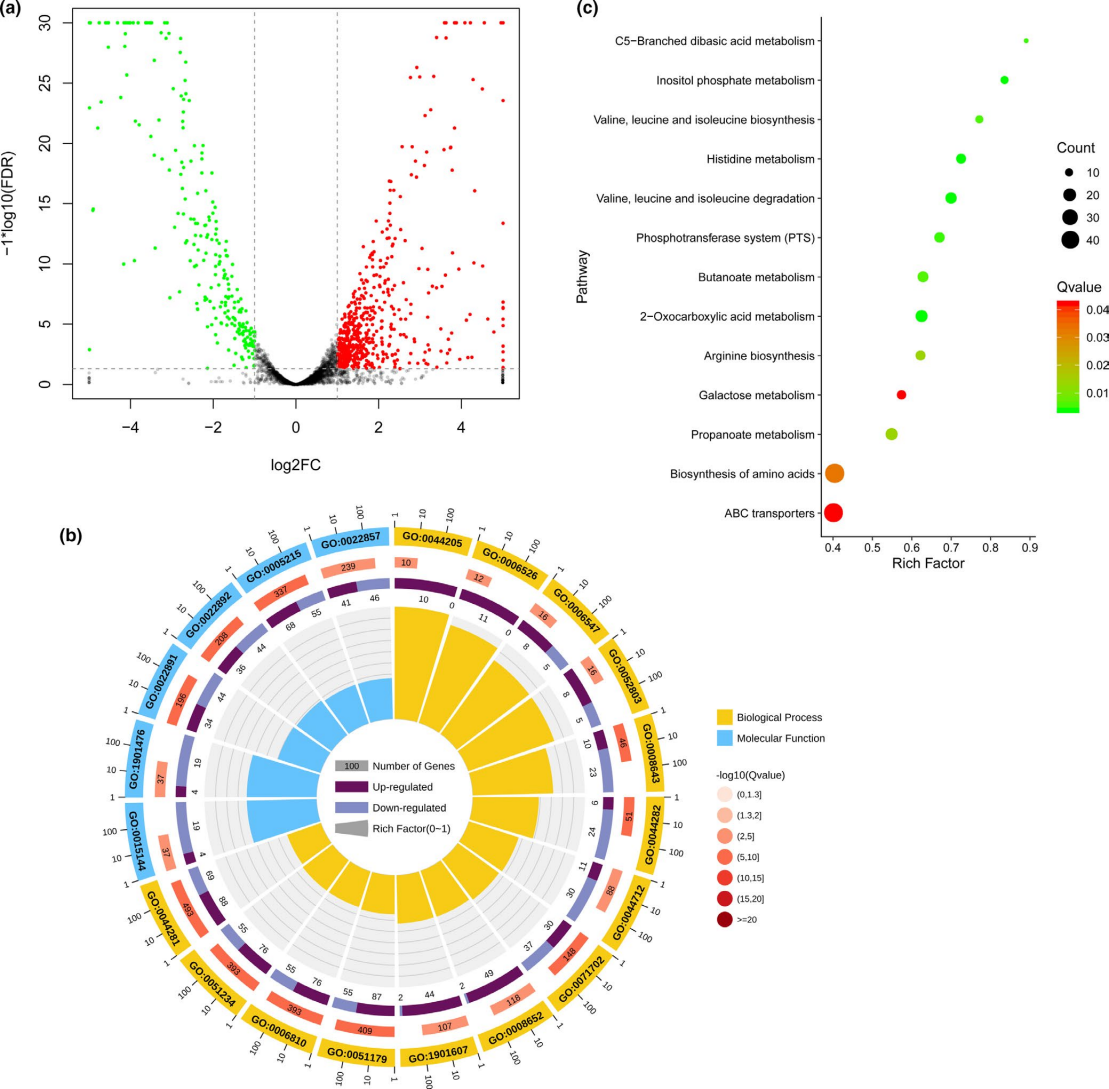
Comparative transcriptomics of *B*. *amyloliquefaciens* strain GKT04 during FOC4 inhibition. (a) A volcano plot of the gene expression pattern in strain GKT04 following its interaction with FOC4. Samples cultured in PDA medium alone were used as controls (CK). The black, red, and green points represent genes with no difference in expression, upregulated genes, and downregulated genes, respectively (FDR ≤0.05, Benjamini and Hochberg adjustment). (b) GO enrichment analysis of the differentially expressed genes (DEGs). The outer ring represents the number of top 20 enriched GO terms, and different colors represent different ontologies. Ring 2 represents gene counts in the whole genome background, with the color changing based on the Q‐value of the enrichment analysis. Ring 3 represents the counts of upregulated and downregulated genes in the corresponding GO term. Ring 4 represents the rich factor (the number of DEGs divided by the number of background genes in the corresponding term). (c) Enrichment of DEGs in the KEGG pathway

Considering that the inhibitory activity was extremely high on Day 3 (Figure 1B), we collected samples on Day 3 and performed RNA‐Seq to investigate the key genes of strain GKT04 that were involved in the inhibition of FOC4. GKT04 interaction with FOC4 resulted in 817 DEGs in GKT04, representing 19.99% of all protein‐coding genes in this bacterial strain (Figure 4A). Of these, 575 genes were significantly upregulated, while 242 genes were significantly downregulated compared to the CK samples (Table S1: https://doi.org/10.6084/m9.figshare.14403971). Approximately 96.70% (790/817) of the DEGs were coding genes, and only a few (3.30%, 27/817) were located on the plasmid. These DEGs were mainly enriched in molecular function categories such as localization, transport, and small molecule metabolic processes and the biological process category of transporter activity (Figure 4B, Table S2: https://doi.org/10.6084/m9.figshare.14403971). However, the functional enrichment patterns of the upregulated and downregulated genes differed. The upregulated genes were specifically associated with functional categories of “*de novo*” UMP biosynthetic and arginine biosynthetic processes and were dominant in the functional categories of cellular amino acid biosynthetic and alpha‐amino acid biosynthetic processes (Figure 4B), indicating that such functional proteins might play important roles in the inhibition of FOC4.

**FIGURE A3:**
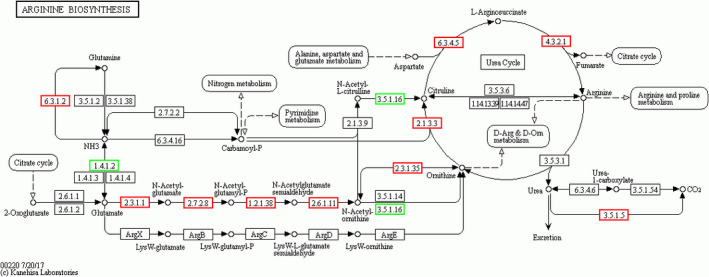
The impact of differentially expressed genes on the arginine biosynthesis pathway. Red indicates that the gene is upregulated and green indicates that the gene is downregulated in *B*. *amyloliquefaciens* GKT04 during FOC4 inhibition

**FIGURE A4:**
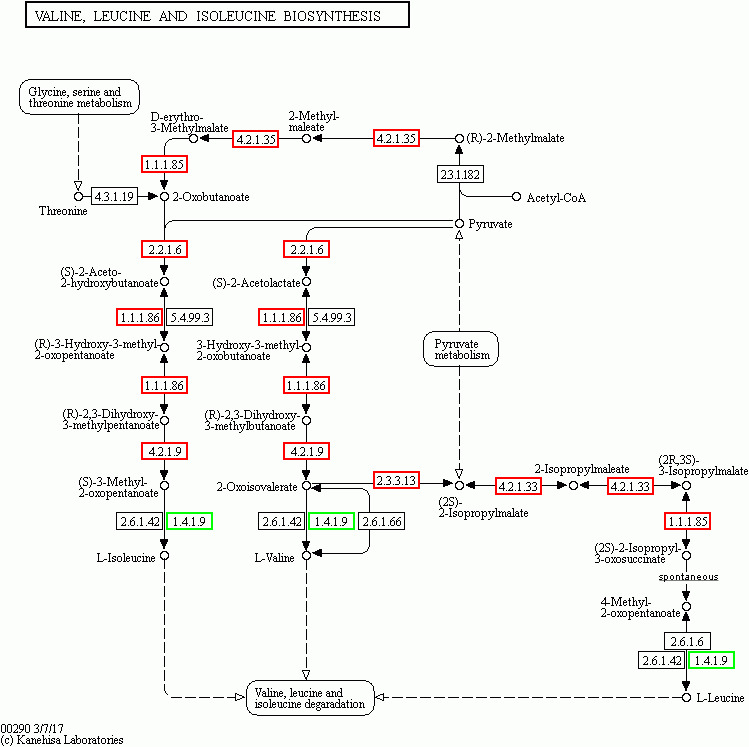
The impact of differentially expressed genes on valine, leucine, and isoleucine biosynthesis pathways. Red indicates that the gene is upregulated and green indicates that the gene is downregulated in *B*. *amyloliquefaciens* GKT04 during FOC4 inhibition

**FIGURE A5:**
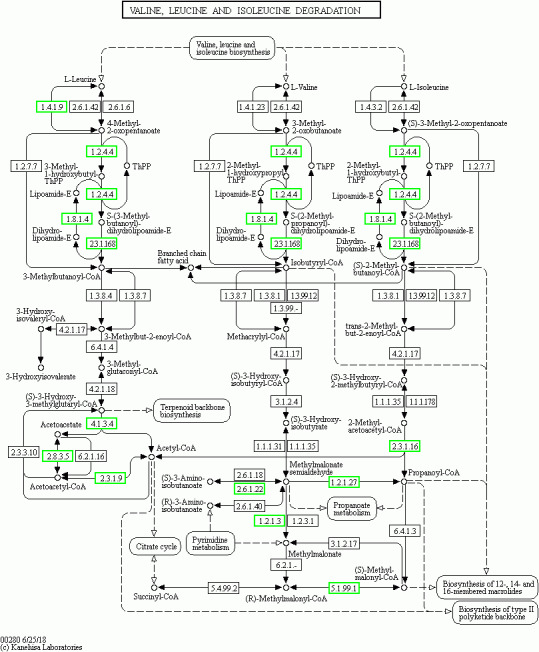
The impact of differentially expressed genes on valine, leucine, and isoleucine degradation pathways. Red indicates that the gene is upregulated and green indicates that the gene is downregulated in *B*. *amyloliquefaciens* GKT04 during FOC4 inhibition

**FIGURE A6:**
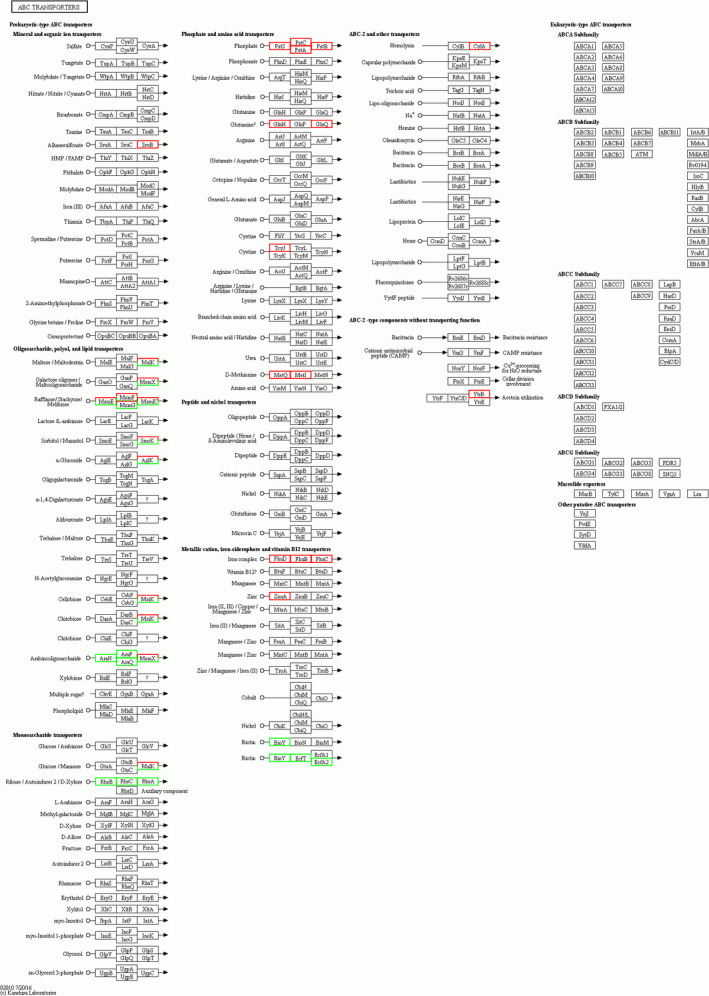
The impact of differentially expressed genes on the ABC transporter pathway. Red indicates that the gene is upregulated and green indicates that the gene is downregulated in *B*. *amyloliquefaciens* GKT04 during FOC4 inhibition

The DEGs were annotated in the KEGG database to identify the pathways involved in the antifungal processes of strain GKT04. Analysis revealed the enrichment of 13 pathways for DEGs, most of which were associated with amino acid and carbohydrate metabolism (Figure 4C). Notably, the DEGs showed an increased abundance of amino acids in the strain GKT04 that interacted with FOC4, with enrichment in the biosynthesis of amino acids, in particular, arginine, valine, leucine, and isoleucine (Figures A3 and A4). Correspondingly, the valine, leucine, and isoleucine degradation pathways were downregulated (Figure A5). In addition, genes associated with phosphate and amino acid transporters were significantly upregulated in the ABC transporter pathway (Figure A6).

### 3.5 Gene regulatory networks of biosynthesis components in strain GKT04

**FIGURE 5:**
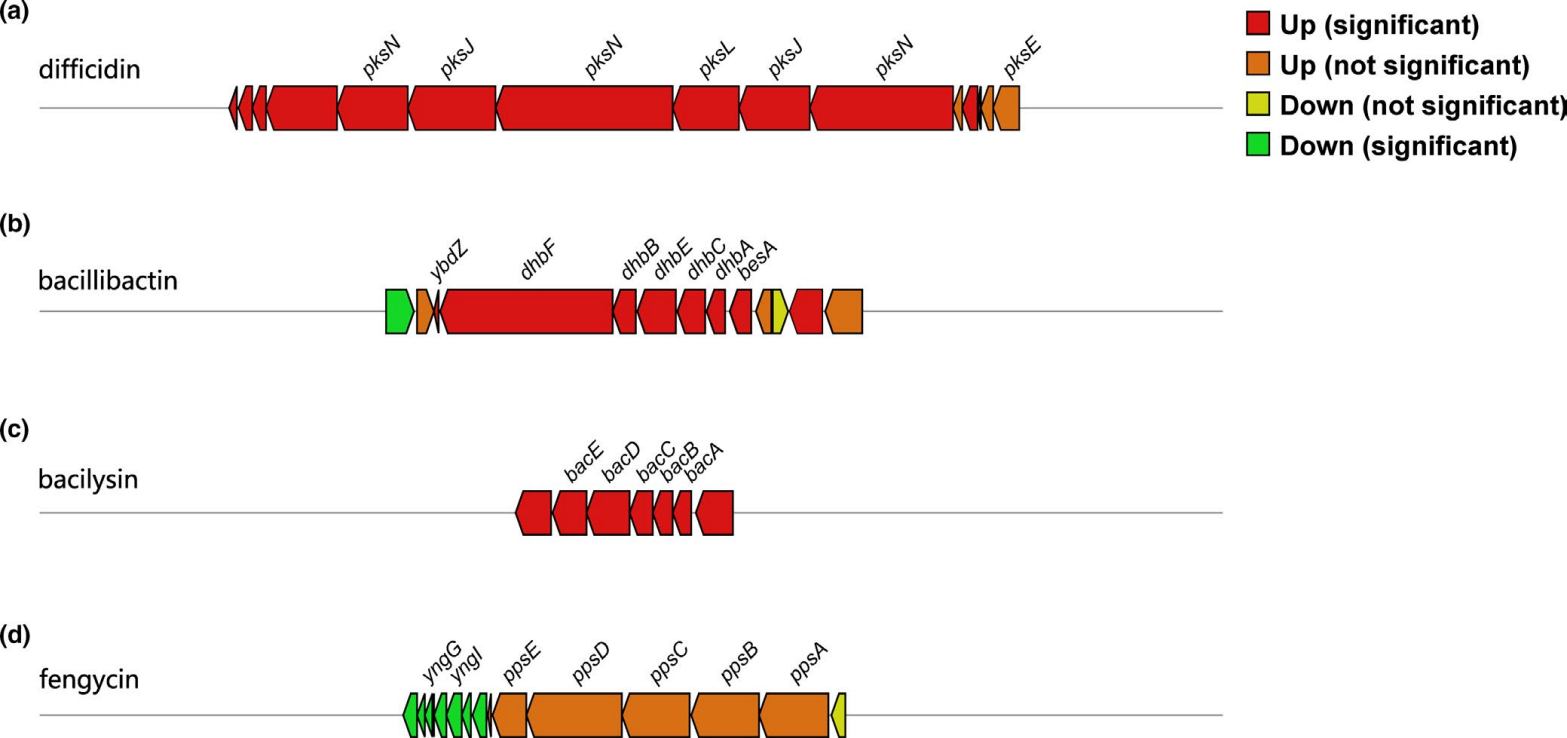
Genomic architecture of the gene clusters potentially encoding difficidin (a), bacillibactin (b), bacilysin (c), and fengycin (d). The genes are marked with different colors representing their differential expression conditions in *B*. *amyloliquefaciens* strain GKT04 during FOC4 inhibition

We further analyzed whether any secondary metabolite biosynthetic gene clusters were differentially expressed during FOC4 inhibition. Of all the predicted gene clusters, four harbored DEGs in more than half of their total genes (Figure 5). Notably, almost all DEGs in gene clusters 1 (producing difficidin), 2 (producing bacillibactin), and 3 (producing bacilysin) were upregulated (Figure 5A‐C), indicating that the elevated production of these antibiotic compounds might play a key role in the antifungal activity of GKT04 against FOC4. However, the core biosynthetic genes in cluster 8 (producing fengycin) showed insignificant upregulation, and all the corresponding regulatory genes were significantly downregulated (Figure 5D). Although fengycin has been shown to inhibit the growth of FOC4 (Tian et al., 2020), the apparent non‐induction of its biosynthesis during the interaction between GKT04 and FOC4 shows that it does not play a role in this process.

**FIGURE 6:**
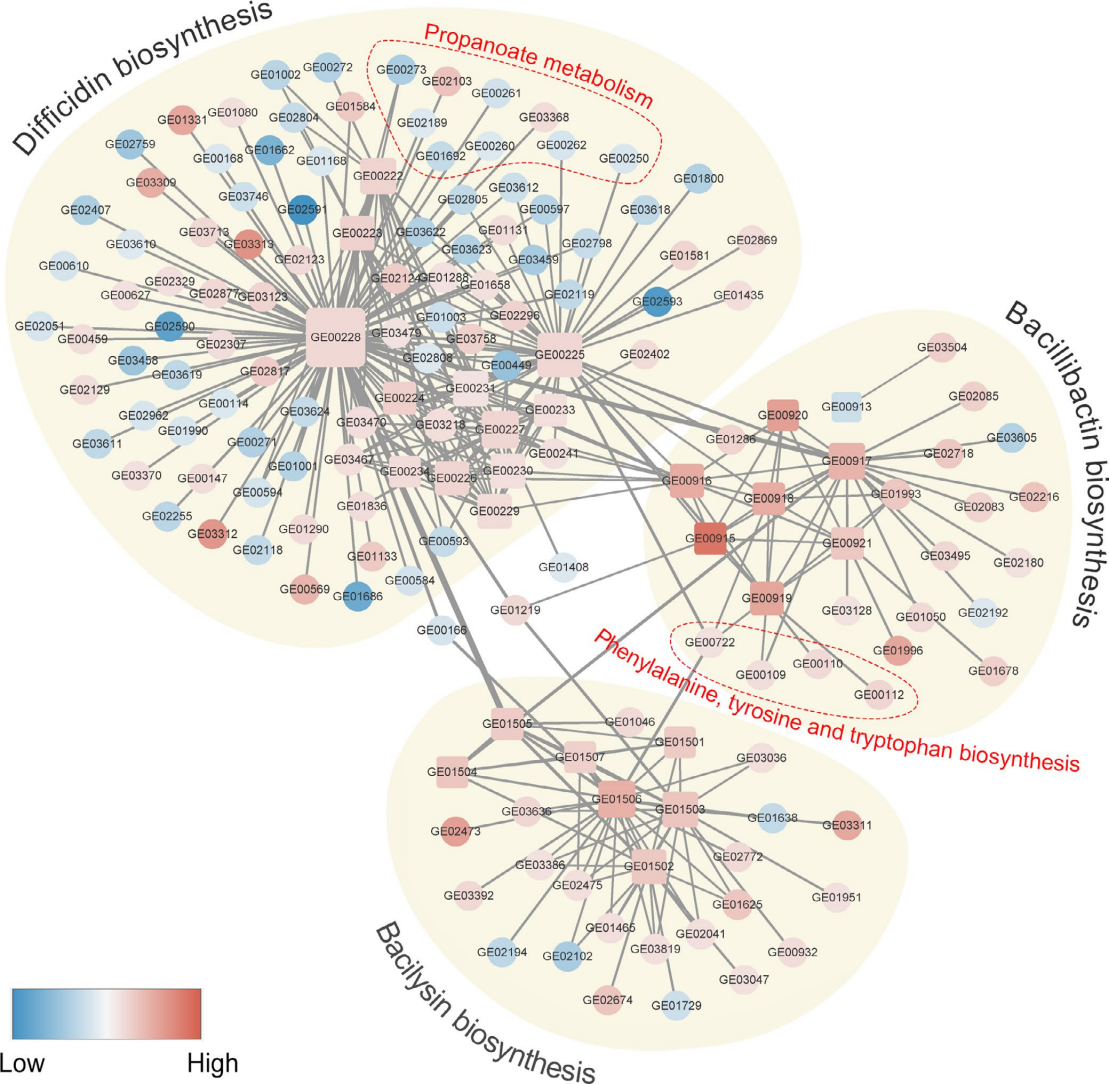
Protein–protein interaction network of DEGs in gene clusters responsible for difficidin, bacillibactin, and bacilysin biosynthesis, as well as other DEGs. Genes in the square are DEGs in gene clusters, and genes in the circle are other DEGs. The gene color is based on the expression level in *B*. *amyloliquefaciens* strain GKT04 during FOC4 inhibition. The thickness of lines between any two genes corresponds to the interaction score between those two genes

The PPI network of DEGs in gene clusters 1, 2, and 3 and other DEGs was analyzed using the STRING database. The results showed that the three clusters were regulated relatively independently of each other (Figure 6). Although all genes in the difficidin production cluster were significantly upregulated, several other genes were downregulated, such as acetoin dehydrogenase (GE02590, GE02591, and GE02593), histidine ammonia‐lyase *HutH* (GE01662), and inositol 2‐dehydrogenase (GE01686) components. In addition, the difficidin production‐related gene cluster interacted with multiple genes from the propanoate metabolism pathway, whose expression was most significantly downregulated, implying a negative interaction between propanoate metabolism and difficidin biosynthesis. Several upregulated genes from phenylalanine (Phe), tyrosine (Tyr), and tryptophan (Trp) biosynthesis pathways interacted with the bacillibactin production‐related gene cluster, indicating potential co‐regulation of biosynthesis of these amino acids and bacillibactin. Notably, one gene in this pathway (GE00722, annotated as Chorismate mutase *AroA*) also interacted with difficidin and bacilysin production gene clusters. Overall, these results show that the gene regulatory networks for antifungal metabolite biosynthesis, including those for difficidin, bacillibactin, and bacilysin, are present in the strain GKT04.

## 4 DISCUSSION

Endophytic bacteria are widely present in plants. They possess rich biological diversity and have positive effects on the host plants. One of the current major challenges in agriculture is obtaining higher crop yields. Environmental conditions, cultivar quality, and plant diseases greatly affect plant productivity. Some endophytic *Bacillus* species are already being used as complementary, effective, and safe alternatives for crop management. Endophytic *Bacillus* species provide plants with a wide range of benefits, including protection against phytopathogenic microorganisms, insects, and nematodes; disease resistance; and promotion of plant growth without causing damage to the environment (Radhakrishnan et al., 2017; Ralf et al., 2018; Xia et al., 2015). We previously reported that the endophytic bacterium *B*. *amyloliquefaciens* GKT04, isolated from banana roots, showed remarkable antagonism to FOC4 in vitro and conferred disease resistance to potted plants (Tian et al., 2018). Moreover, the fermentation supernatant containing GKT04 significantly inhibited colony growth and FOC4 spore germination. Fengycins and bacillomycin that have previously been isolated from the supernatant of GKT04 showed obvious antifungal effects (Tian et al., 2020). In the present study, we characterized the genomic features of this strain and investigated its gene expression patterns in response to FOC4.

ANI analysis revealed that GKT04 was highly homologous to strains KHG19 and SH‐B74 (Figure 2). SH‐B74 produces the cyclic lipopeptide plipastatin A1, which has excellent in vitro ability to suppress the germination of *B*. *cinerea* conidia, the causal agent of gray mold disease in tomato (Ma & Hu, 2018). The evolutionary similarity between GKT04 and this strain implies the potential ability of GKT04 to produce antifungal lipopeptides.

The annotation of *B*. *amyloliquefaciens* GKT04 proteins revealed that strain GKT04 exhibited strong metabolic activity in several aspects (Figure A1). Both antibiotic synthesis and bacterial reproduction occur through complex metabolic processes. For example, the transport and metabolism of amino acids are involved in the formation of antifungal lipopeptides, which are secondary metabolites containing peptides. Bacterial cell division requires nucleic acids, carbohydrates, inorganic ions, and other biochemical components. The transport and metabolism of these substances, and processes such as cell wall synthesis, are important for bacterial cell division (Mahone & Goley, 2020). In this study, several proteins associated with cell wall/membrane/envelope biogenesis were identified, suggesting that the GKT04 strain has a potent ability for cell division and the potential to achieve large biomass for antifungal action during the biocontrol process.

Annotation using antiSMASH revealed that strain GKT04 might have multiple gene clusters that encode polyketides, siderophores, and lipopeptides, suggesting that GKT04 may antagonize fungi by producing these secondary metabolites. Polyketides, siderophores, and lipopeptides are common secondary metabolites that have demonstrated antifungal activity. Polyketides in PKS are synthesized from acyl‐CoA precursors, such as malonate and methyl malonate (Smith & Tsai, 2007). Siderophores and lipopeptides were synthesized using NRPS. Although siderophores exhibit different configurations and properties, all siderophores share a conserved structure that consists of a functional unit that ligates with molecules such as transferrin and lactoferrin. Typically, they possess a peptide backbone that interacts with the outer membrane receptors present on the cell surface (Marathe et al., 2015). The lipopeptides of *Bacillus* are small metabolites that contain a cyclic structure formed by 7–10 amino acids (including 2–4 d‐amino acids) and a *β*‐hydroxy fatty acid with 13–19 C atoms (Zhao et al., 2017). They are highly effective against fungi, bacteria, and viruses. Different *B*. *amyloliquefaciens* contain different lipopeptides, and even the lipopeptides produced by the same strain are complex compounds consisting of several homologs with similar structures (Peypoux et al., 1999). Based on the annotation of gene clusters, the strain GKT04 was shown to produce a variety of lipopeptides, including bacilysin, surfactin, bacillomycin D, and fengycin (Table 2).

RNA‐Seq identified 575 genes that were upregulated and 242 genes that were downregulated in GKT04 during its inhibition of FOC4. Although the functional enrichment patterns between the upregulated and downregulated genes were different, both pointed to the accumulation of valine, leucine, and isoleucine, since the biosynthesis of these amino acids was upregulated, while their degradation was downregulated. There is inevitable competition between the biosynthesis of antibiotic substances and biomass formation. Both biosynthesis and proliferation of antibiotic secondary metabolites require energy and substances such as amino acids and fatty acids. Amino acids, which are intrinsic components of lipopeptides and siderophores, are simultaneously used in the formation of cell structural components as well as various enzymes. We suspect that the increased valine, leucine, and isoleucine concentrations are beneficial for the biosynthesis of antifungal metabolites. These amino acids may act as additional resources for the synthesis of peptide‐containing antifungal metabolites or enzymes. In addition, some upregulated genes associated with phosphate and amino acid transporters in the ABC transporter pathway were identified. Previous studies have shown that the knock‐out of amino acid transporter genes can inhibit the biosynthesis of lipopeptide polymyxin B‐type antibiotics (Shaheen et al., 2011). Thus, the upregulation of these genes may indicate the enhancement of lipopeptide antibiotics.

Most genes in the clusters producing difficidin, bacillibactin, or bacilysin were upregulated (Figure 5A‐C), indicating that FOC4 inhibition by GKT04 relies mainly on increasing the biosynthesis of the three antifungal metabolites in *Bacillus* spp. Polyketide difficidin and lipopeptide bacilysin produced by *B*. *amyloliquefaciens* are efficient in controlling fire blight disease (Chen et al., 2009). The siderophore bacillibactin of *B*. *amyloliquefaciens* SQR9 was upregulated when SQR9 was combined with multiple fungi, including *Fusarium solani* (Li et al., 2014). However, another component, fengycin, which was previously shown to be constitutively produced by GKT04 and effective in inhibiting FOC4 growth (Tian et al., 2020), showed no significant change in its biosynthesis. This indicates that fengycin biosynthesis might play a basic antifungal role and not directly respond to the interaction between GKT04 and FOC4.

Further analysis of gene interactions in the three upregulated clusters revealed that the three relatively independent clusters were also related to each other. Many genes that interacted with the genes involved in difficidin production were downregulated during this antifungal process, indicating the potential existence of multiple negative regulators of difficidin biosynthesis. Among the downregulated products, acetoin dehydrogenase catalyzes the oxidative decarboxylation of 2‐oxoacids to their respective acyl‐CoAs in eukaryotes and aerobic bacteria. *AcoB* (GE02591) is a pyruvate dehydrogenase beta subunit. The pyruvate dehydrogenase complex converts pyruvate to acetyl‐CoA, thus linking glycolysis with tricarboxylic acid (TCA) (Payne et al., 2010; Perham, 2000). Glycolysis and the TCA cycle are important energy metabolism pathways that produce energy for organisms. Several genes involved in the propanoate metabolism pathway were also downregulated in this cluster. Propionyl‐CoA is formed during the oxidation of odd‐carbon‐numbered fatty acids, oxidative degradation of the branched‐chain amino acids valine and isoleucine, and from the carbon skeletons of methionine, threonine, thymine, and cholesterol (Rosenberg & Lawson, 1982). We suspect that the downregulation of valine and isoleucine degradation may account for the decreased propionate metabolism. Although propionate has specific functions in various organisms, much of it is catabolized. The product of microbial metabolism by using propionate is usually succinate or acetate, which can enter the TCA cycle. The propionate‐to‐succinate pathway occurs in many genera of microorganisms, including *Rhodospirillum*, *Propionibacterium*, and *Mycobacterium*. In *Prototheca zopfi* and *Clostridium kluyveri*, the catabolism of propionate eventually leads to the production of acetyl‐CoA in the TCA cycle via a 3‐hydroxypropionate intermediate (Haase et al., 1984; Halarnkar & Blomquist, 1989; Wegener et al., 1968). These results indicate that multiple genes associated with energy metabolism negatively regulate the biosynthesis of difficidin.

Most genes that interact with bacillibactin or the bacilysin production‐related gene cluster were upregulated, indicating the presence of a series of potential positive bacillibactin or bacilysin regulators. Some upregulated genes in Phe, Tyr, and Try biosynthesis pathways interacted with the bacillibactin production‐related gene cluster. In microorganisms, the shikimate pathway branches at many points. It is used to synthesize the three proteinogenic aromatic amino acids (Phe, Tyr, and Trp), folate coenzymes, benzoid and naphtoid quinones, and a broad range of mostly aromatic secondary metabolites, including some siderophores. The last common branch point of these compounds is chorismate. Chorismate is a common precursor in the biosynthesis of Phe, Tyr, and Try, as well as siderophores (Dosselaere & Vanderleyden, 2001). The four genes that clustered into Phe, Tyr, and Try biosynthesis pathways are referred to as chorismate metabolism‐related genes (Table S1: https://doi.org/10.6084/m9.figshare.14403971). They may interact with isochorismatase *dhbB* (GE00917), enterobactin synthase subunit EdhbCE (GE00918), and/or isochorismate synthase *dhbC* (GE00919) in a coordinated manner to promote the biosynthesis of the siderophore bacillomycin. In addition, chorismate mutase *AroA* (GE00722), one of the genes in this pathway, also interacted with difficidin and bacilysin production gene clusters, while GE00109, GE00110, and GE00112 did not. Although *AroA* is the only gene in Phe, Tyr, and Try biosynthesis pathways that interacted with difficidin and bacilysin production gene clusters, multiple genes involved in chorismate metabolism also interacted with at least one of these clusters, for example, GE00917 and GE00918. These results indicate that chorismate metabolism affects the secondary metabolites polyketide difficidin, siderophore bacillibactin, and lipopeptide bacilysin. The effects of chorismate metabolism‐related genes on polyketide and lipopeptide production have also been reported in previous studies. A chorismatase/3‐hydroxybenzoate synthase gene was identified in an orphan type I polyketide synthase gene cluster in *Streptomyces* sp. LZ35 (Jiang et al., 2013). In addition, chorismate mutase *PheB* was involved in regulating the production of bacillomycin and fengycin by *B*. *amyloliquefaciens* Q‐426 (Zhao et al., 2015).

In summary, through a combined analysis of genomic and transcriptomic sequence data, we demonstrated that the biosynthesis of difficidin, bacillibactin, and bacilysin is enhanced during FOC4 inhibition by *B*. *amyloliquefaciens* GKT04. We also presented a regulatory network of gene interactions involved in the biosynthesis of these antifungal metabolites. Several genes involved in amino acid biosynthesis, chorismate metabolism, and propanoate metabolism pathways, among others, may be involved in regulating the synthesis of antifungal compounds. These results broaden our understanding of the antifungal mechanism of *B*. *amyloliquefaciens*. However, the specific mechanism requires further verification.

## CONFLICT OF INTEREST

None declared.

## AUTHOR CONTRIBUTIONS

**Dandan Tian:** Conceptualization (lead); Data curation (equal); Investigation (lead); Writing‐original draft (lead); Writing‐review & editing (lead). **Xiupeng Song:** Conceptualization (equal); Investigation (equal); Methodology (lead); Writing‐original draft (equal); Writing‐review & editing (equal). **Chaosheng Li:** Conceptualization (equal); Funding acquisition (lead); Project administration (lead); Supervision (equal); Writing‐original draft (equal); Writing‐review & editing (equal). **Wei Zhou:** Data curation (equal); Formal analysis (equal); Visualization (equal). **Liuyan Qin:** Data curation (equal); Formal analysis (equal); Software (equal). **Liping Wei:** Data curation (equal); Resources (equal). **Di Wei:** Data curation (equal); Resources (equal). **Sumei Huang:** Formal analysis (equal); Resources (equal). **Baoshen Li:** Formal analysis (equal); Validation (equal). **Quyan Huang:** Data curation (equal); Software (equal). **Shengfeng Long:** Investigation (equal); Validation (equal). **Zhangfei He:** Formal analysis (equal); Visualization (equal). **Shaolong Wei:** Funding acquisition (equal); Methodology (equal); Project administration (equal); Writing‐original draft (equal); Writing‐review & editing (equal).

## ETHICS STATEMENT

None required.

## Data Availability

All data are provided in full in this paper except the genomic and transcriptomic data, which are available at NCBI database under BioProject PRJNA716416 (https://www.ncbi.nlm.nih.gov/bioproject/PRJNA716416) and in figshare (https://doi.org/10.6084/m9.figshare.14403971).
